# Account of a Diseased Testicle, Successfully Removed at an Advanced Stage

**Published:** 1810-01

**Authors:** John Taunton

**Affiliations:** Surgeon to the City and Finsbury Dispensaries, &c.


*Account of a diseased Testicle, successfully removed at an advanced Stage;* by Mr. JOHN TAUNTON, Surgeon to the City and Finsbury Dispensaries, &c.

S. S. æt. 35, applied to me for advice June 9, 1809. He had perceived a small swelling at the bottom of the scrotum about twenty months previous to my seeing him; this

---

this increased gradually, but was not attended with pain till last winter, when the cold affected it considerably. The tumour was of a pyriform figure, having a smooth surface, elastic, and appearing to contain a fluid. The spermatic cord, where it entered the ring, was perfectly free from disease, but the integuments covering the tumour were much inflamed and very painful. He was of a spare habit of body. A fomentation of poppies was directed, with internal remedies varied according to circumstances.

June 10. I proposed puncturing the tumour with a view to ascertain the state of the testicle, but under the full impression that castration would be necessary. On introducing the trocar, only about half a table spoonful of a brownish glutinous liquid passed the canula. This operation did not give more pain than was occasioned by the increased sensibility of the integuments. The remedies were continued, with the external use of aq. litharg. acet. comp.

11th. This lotion had procured much more ease than the poppy fomentation, by removing the local inflammation. In consultation with Mr. Bartlett, it was now agreed that castration was the only remedy. This was performed by placing the patient on a table, and including in the incision a large portion of integuments on the anterior and inferior part of the tumour. The external pudendal artery was the only vessel which required to be tied till the whole was removed. The spermatic process was tied by separating the vas differens, and passing very firmly round the remaining part a ligature, which did not produce much pain, nor was any felt on dividing it. About eight vessels required to be taken up, and these principally from the septum scroti. The operation occupied much time, on account of the number of vessels taken up, and also from the great danger of wounding the skin and septum scroti in so extensive a dissection. A great quantity of blood was lost during the operation, but the pulse remained strong and regular, not exceeding 80 in the minute. The wound was dressed with four sutures, lint, and adhesive plaster. The patient bore the operation well.

At 3 h. P. M. four hours after the operation, the pulse was 120, weak, and irregular; the countenance fallen; he complained of sickness, and smarting pains in the part. Much blood had been lost, but the hæmorrhage was apparently stopped. He could not turn on the side; and lying on his back distressed him much. From the great debility bility I did not think proper to change the position of his body, but ordered cloths, wet with the aqua litharg. acet. comp. to be applied over the dressing to the scrotum.

At 7 P. M. pulse 130; face and lips pale from the loss of blood; complained much of sickness; the lotion had eased the pain. On removing the cloths and coagulum, it appeared there had been a trifling hæmorrhage since the last visit, but which was now completely stopped.— ℞. Aq. menth. sat. aq. ammon. acet. aa. cochl. duo sextis horis sumenda. Lotion repeated.

June 12. At 5 h. A. M. the countenance was much improved; pulse 100, and stronger; he complained of having had but little rest during the night, and that the medicine produced sickness. He now took an infusion of cascarilla with nitrous acid, and the lotion repeated.

4 P. M. Much better; pulse 94; free from pain, making water easily, but no alvine evacuation. The wound looked well at the upper part, where the lint was removed; the lotion contributed greatly to his ease; he had obtained some sleep; was in a gentle perspiration; and the medicine agreed with the stomach.

The tumour weighed upwards of three pounds; there was contained in the tunica vaginalis, at the upper part, about six ounces of a straw-coloured fluid, which coagulated on the application of heat; the bulk of the tumour was formed by an enlargement of the body of the testicle, which was composed of a reddish brown substance of a soft pulp-like structure, much resembling the fungus hematoides of Hay. The centre of the testicle was most pulpy, but nothing appeared like hydatids or separate cysts; neither was it produced by an effusion of blood, but evidently a morbid growth of the part. In this case, the water contained in the tunica vaginalis, at the upper part, gave an elastic feel and an undulating motion to every part of the tumour.

June 13. Some smarting pain had been experienced in the wound, in consequence of the lotion being omitted from the inattention of the patient or those about him; but the wound looked well; pulse 90, which, on rising up in bed, increased suddenly to 120, rather irregular, but moderately full. He had two evacuations by stool on the preceding evening; the medicine agreed with the stomach, and he was in every respect going on well.

14. Felt somewhat depressed, but easy with the application of the lotion to the part; pulse 80, but on rising up up in bed increased to about 96. The wound looked well, and the appetite began to return. Medicine repeated.

June 15 and 16. Pulse 72; free from pain; one of the ligatures was removed, and the wound dressed with the ung. hydr. nitrico-oxydi. Medicine repeated.

17, 18, 19. Another ligature was removed; suppuration had taken place to a small extent upon the chord at the part where it was tied. The patient much stronger, and occasionally sat up in bed. Medicine repeated.

20 and 21. Rather low; removed all the ligatures except the one on the chord. Medicine repeated.

22, 23. Removed the ligature on the chord, and carried a strap of adhesive plaster around the scrotum to keep the parts in contact. The dressings were applied over the whole.

29. The dressings continued, and the patient walked out daily.

He is at this time in a perfect state of health, and follows his accustomed employment.

On the first examination of this patient, the integuments had a livid appearance, similar to that which precedes gangrene; so much so, that one professional gentleman, to whom he applied the day before, considered it a lost case, and would not attend him. This local inflammation was relieved more by cold than by warm applications, which were first used. The subsequent pain and inflammation of the wound were invariably mitigated to an extent I have rarely seen by the solution of lead.

The extreme debility of this patient at the time I first visited him, rendered the issue of the operation doubtful; but as the only mode of affording him even a chance of life, it was preferred with his full approbation. Altho' I should certainly prefer operating at an early period of the disease, in those cases where an operation is absolutely necessary, yet I think the patient ought not to be deserted even at a very late period, from the apprehension of failure and bringing the operation into disrepute. The above conclusion is fully warranted by the result of some cases formerly published.

*Greville Street, Hatton Garden, Nov. 22, 1809.*

